# Meta‐analysis of the quantitative trait loci associated with agronomic traits, fertility restoration, disease resistance, and seed quality traits in pigeonpea (*Cajanus cajan* L.)

**DOI:** 10.1002/tpg2.20342

**Published:** 2023-06-16

**Authors:** Priyanka Halladakeri, Santosh Gudi, Sabina Akhtar, Gurjeet Singh, Dinesh Kumar Saini, Harshavardan J. Hilli, Amar Sakure, Sneha Macwana, Reyazul Rouf Mir

**Affiliations:** ^1^ Department of Genetics and Plant Breeding Anand Agricultural University Gujarat India; ^2^ Department of Plant Breeding and Genetics Punjab Agricultural University Ludhiana Punjab India; ^3^ College of Education American University in the Emirates Dubai UAE; ^4^ Department of Agricultural Biotechnology Anand Agricultural University Gujarat India; ^5^ Division of Genetics and Plant Breeding Faculty of Agriculture SKUAST‐Kashmir Wadura India

## Abstract

A meta‐analysis of quantitative trait loci (QTLs), associated with agronomic traits, fertility restoration, disease resistance, and seed quality traits was conducted for the first time in pigeonpea (*Cajanus cajan* L.). Data on 498 QTLs was collected from 9 linkage mapping studies (involving 21 biparental populations). Of these 498, 203 QTLs were projected onto “PigeonPea_ConsensusMap_2022,” saturated with 10,522 markers, which resulted in the prediction of 34 meta‐QTLs (MQTLs). The average confidence interval (CI) of these MQTLs (2.54 cM) was 3.37 times lower than the CI of the initial QTLs (8.56 cM). Of the 34 MQTLs, 12 high‐confidence MQTLs with CI (≤5 cM) and a greater number of initial QTLs (≥5) were utilized to extract 2255 gene models, of which 105 were believed to be associated with different traits under study. Furthermore, eight of these MQTLs were observed to overlap with several marker‐trait associations or significant SNPs identified in previous genome‐wide association studies. Furthermore, synteny and ortho‐MQTL analyses among pigeonpea and four related legumes crops, such as chickpea, pea, cowpea, and French bean, led to the identification of 117 orthologous genes from 20 MQTL regions. Markers associated with MQTLs can be employed for MQTL‐assisted breeding as well as to improve the prediction accuracy of genomic selection in pigeonpea. Additionally, MQTLs may be subjected to fine mapping, and some of the promising candidate genes may serve as potential targets for positional cloning and functional analysis to elucidate the molecular mechanisms underlying the target traits.

## Core Ideas

- Meta‐analysis of 203 QTLs predicted 34 meta‐QTLs (MQTLs) associated with several important traits in pigeonpea.
- Candidate gene analysis identified 105 candidate genes within the MQTL regions.
- Three promising MQTLs (viz., CC_MQTL2.3, CC_MQTL3.2, and CC_MQTL11.1) were recommended for the pigeonpea breeding.

**Abbreviations**
CG: candidate gene
CI: confidence interval
GS: genomic selection
GWAS: genome‐wide association studies
MAB: marker‐assisted breeding
MQTL: meta‐QTL
MTA: marker‐trait association
QTLs: quantitative trait loci

## 1 INTRODUCTION

Pigeonpea (*Cajanus cajan* L.) is a diploid legume crop with a genome size of 833.1 Mb (Varshney et al., 2012). It is the sixth most important grain legume crop grown, specifically, by resource‐constrained farmers in the semiarid and subtropical regions of Asia and Africa. Pigeonpea is a climate‐resilient and less resource‐demanding legume crop with a rich source of proteins, essential amino acids, and minerals (Jorrin et al., 2021; Singh, Gudi, et al., 2022).

Decades of breeding efforts, including CMS‐based hybrid technology (for exploitation of heterosis), resulted in the development of a number of high‐yielding varieties in pigeonpea (Sharma et al., 2019). Despite these progresses, the productivity of pigeonpea is not sufficient to fulfill the food requirements of an ever‐increasing human population (Bohra et al., 2020). This is owing to the presence of inherent physiological and genetic constraints, such as indeterminate growth, delayed maturity, and low selection efficiency, which hampered its breeding efforts (Saxena et al., 2021). Furthermore, pigeonpea production faces a number of biotic stresses such as Fusarium wilt and sterility mosaic disease (SMD), which ultimately affect the production potential of this crop (Singh, Singh, et al., 2020). Plant breeders are concerned with combining multiple yield‐related traits (Singh, Gudi, et al., 2022; Singh, Kaur, et al., 2022). A paradigm shift may occur in the near future if the conventional breeding (pre‐breeding, and alien gene introgression) approaches are integrated with modern breeding tools (such as genomics, phenomics, speed breeding, genetic engineering, haplotype‐based breeding, allele mining, and genome editing) (Bakala et al., 2020; Gudi, Kumar, et al., 2022).

Unraveling the genomic regions associated with the quantitative traits and their utilization in breeding programs may help in alleviating these production constraints (Ahmar et al., 2020; Lu et al., 2018). The discovery of molecular markers and quantitative trait loci (QTLs) mapping techniques have aided in dissecting the genetic basis of quantitative traits. QTL mapping also provides a foundation of marker‐assisted selection (MAS) that expedites the breeding process through indirect selection. Though the first QTL mapping study using molecular markers was carried out in 1988 in tomato (Paterson et al., 1988), its application was delayed until 2010 in pigeonpea. This might be due to the lack of information on molecular markers and linkage maps. The first QTL mapping in pigeonpea was reported for SMD in 2011 by developing intraspecific genetic maps by utilizing two F_2_ populations (Gnanesh et al., 2011). Since then, several QTL studies have been conducted in pigeonpea by using F_2_, recombinant inbred lines (RILs), and backcross (BC) populations. These efforts led to the discovery of a number of QTLs associated with different traits, including fertility restoration, Fusarium wilt, SMD, plant height, days to flowering, growth habit, branching pattern, pod yield, protein content, seed weight, and seed yield (Bohra et al., 2012, 2020; Kumawat et al., 2012; Obala et al., 2020; Saxena et al., 2017, 2018). However, the results of QTL mapping studies based on biparental populations are strongly influenced by the type and density of the marker sets used, choice of parents, type and size of the mapping populations, experimental conditions, and statistical methods employed (Khahani et al., 2021). Furthermore, the majority of the QTLs identified in the biparental mapping populations are minor QTLs with low stability and reliability, making them unsuitable for MAS and positional cloning (Quraishi et al., 2017). Therefore, it is of utmost importance to identify most stable and reliable QTLs with large phenotypic effects. Furthermore, genome‐wide association studies (GWAS) have been considered a powerful technique in discovering the marker‐trait associations (GWAS‐MTAs) in pigeonpea (Patil et al., 2017) and other crops (Saini, Chopra, et al., 2022). In addition, some reports revealed the confirmation of GWAS‐MTAs using QTL mapping studies (Chen et al., 2019; Wu et al., 2021). This implies that combining linkage‐based mapping and GWAS results can be useful in identifying important genomic regions associated with key traits in pigeonpea.

**Core Ideas**

- Meta‐analysis of 203 QTLs predicted 34 meta‐QTLs (MQTLs) associated with several important traits in pigeonpea.
- Candidate gene analysis identified 105 candidate genes within the MQTL regions.
- Three promising MQTLs (viz., CC_MQTL2.3, CC_MQTL3.2, and CC_MQTL11.1) were recommended for the pigeonpea breeding.

The meta‐analysis of QTLs is a powerful approach for compiling information from multiple interval mapping studies by tackling the heterogeneity between studies. It helps in identifying the consensus and robust QTL or meta‐QTL (MQTL) regions with a reduced confidence interval (CI) (Goffinet & Gerber, 2000; Veyrieras et al., 2007). The MQTL analysis not only helps in identifying QTL hotspots but can also imply the existence of pleiotropic traits by creating QTL clusters for several traits (Said et al., 2013). Meta‐analysis can be performed using either MQTL or BioMercator software by formulating and inserting particular sets of algorithms for accurate estimation and recalculation of the genetic position for a specified set of QTLs (Wang et al., 2014). The MQTL analysis has already been conducted in different crops, including wheat (e.g., Gudi, Saini, et al., 2022; Kumar et al., 2021; Pal et al., 2021, 2022; Saini et al., 2021; Saini, Chahal, et al., 2022; Saini, Srivastava, et al., 2022; Tanin et al., 2022), rice (e.g., Sandhu et al., 2021), maize (e.g., Kaur et al., 2021), sorghum (e.g., Aquib & Nafis, 2022), barley (e.g., Zhang et al., 2017), and tomato (e.g., Ayenan et al., 2018). Furthermore, MQTL analysis has also been conducted in some legume crops for different traits such as Fe and Zn content and resistance to white mold in French beans (Izquierdo et al., 2018; Vasconcellos et al., 2017), yield‐related traits, seed protein content and partial resistance to *Aphanomyces* root rot in pea (Hamon et al., 2013; Klein et al., 2020), leaf spot resistance, plant height, seed number, and seed yield in peanut (Chen et al., 2019; Lu et al., 2018; Lv et al., 2018), and—quality traits in soybean (Chen et al., 2021). However, the studies reporting meta‐analysis for QTLs are not available in pigeonpea, and this is believed to be the first report that integrates the QTL information from various studies to identify MQTLs associated with multiple traits in pigeonpea.

In the present study, by using results from previous interval mapping studies (published during 2011–2020), a meta‐analysis was conducted to identify novel MQTLs and candidate genes (CGs) associated with agronomic traits, fertility restoration, disease resistance, and seed quality traits. Furthermore, the findings of the meta‐analysis were also compared with the results of GWAS studies earlier conducted in pigeonpea. Further, by utilizing the synteny and collinearity of pigeonpea with other legumes, such as chickpea, pea, cowpea, and French bean, ortho‐analysis was also carried out to examine the transferability of the generated information across the other legume crops. The results of this study could aid in the identification of diagnostic markers and also help in MQTL‐assisted breeding or genomic selection (GS) to improve targeted traits in pigeonpea.

## 2 MATERIALS AND METHODS

### 2.1 Bibliographic search and collection of QTL information

A systematic bibliographic search was conducted on linkage‐based mapping studies published until 2020 to collect information on QTLs associated with different traits of economic importance in pigeonpea. From each of these studies, information on peak position, CI (95%), flanking markers, LOD scores, *R* ^2^ values (i.e., phenotypic variation explained; PVE) of individual QTLs, and the size and type of the mapping populations utilized for mapping were collected (Tables S1 and S2).

The initial QTLs were mainly associated with a total of 21 different traits. All of them were categorized into the following four major trait groups: (i) *agronomic traits* (viz., plant height, growth habit, primary and secondary branches per plant, days to flowering, cleistogamous flower, days to maturity, pods per plant, seed yield, seed size, shriveled seed, seed length, seed width, seed thickness, and seed weight); (ii) *fertility restoration*; (iii) *disease resistance* (viz., Fusarium wilt and SMD); and (iv) *seed quality traits* (viz., seed protein content, water uptake, and electrical conductivity) (Table S3).

### 2.2 Construction of consensus genetic map

The “LPmerge” package (Endelman & Plomion, 2014) built in RStudio was used to generate the high‐density consensus linkage map by integrating the marker information from the following genetic maps: (i) “Pigeon_pea‐ICP_8863/ICPL_20097‐F2,” (ii) “Pigeon_pea‐TTB_7/ICP_7035‐F2” (Gnanesh et al., 2011), (iii) “Pigeon_pea‐Pusa_Dwarf/HDM04‐1‐F2:3” (Kumawat et al., 2012), (iv) “Pigeon_pea‐ICRISAT_consensus_map‐2012” (Bohra et al., 2012), (v) “Pigeon_pea‐ICPL_88039A/AK_250189R‐BC” (Bohra et al., 2017), (vi) “Pigeon_pea‐NRCPB_consensus_map‐2017” (Arora et al., 2017), (vii) “Axiom Cajanus SNP Array” (Yadav et al., 2019), and (viii) “A 62K genic‐SNP chip array” (Singh, Mahato, et al., 2020). Flanking markers of the initial QTLs obtained from individual linkage mapping studies were also included while developing the consensus linkage map.

### 2.3 QTL projection and Meta‐QTL analysis

Information on the initial QTLs, such as peak position, CI, PVE values, LOD scores, the associated trait, and size of the mapping populations, were used to project QTLs on the consensus map. All available QTLs, irrespective of their contribution to the trait phenotype, were included in the meta‐analysis. When the QTL peak positions were not provided in a particular study, the midpoints of the flanking marker positions were used to calculate them. Similarly, whenever the LOD scores for any of the QTLs were not provided, they were assumed to be 3.0 for the purpose of analysis. Furthermore, for the QTLs with no CI available, the CI (95%) of each initial QTLs was calculated according to its population type and size by using the population‐specific equations: (i) for RIL populations: CI = 163/(population size × *R* ^2^); and (ii) for F_2_ and BC populations: CI = 530/(population size × *R* ^2^), where, 163 and 530 are the population‐specific constants (Darvasi & Soller, 1997; Venske et al., 2019).

Redundant QTLs were commonly discovered in mapping studies that were replicated over locations and years. To avoid giving too much weight to such QTLs in the meta‐analysis, only QTLs explaining the maximum phenotypic variation were included in the study. Then the scaling rule (i.e., comparing the genetic positions of flanking markers present on the linkage maps and consensus map, respectively) was used to determine the QTL positions on the consensus map. The Gaussian distribution encircling the most likely QTL position was used to predict the CI of the projected QTLs. All the QTL projections were performed using the QTLProj tool of BioMercator V4.2 (Sosnowski et al., 2012).

The Veyrieras two‐step algorithm, which is included in the BioMercator v4.2 software, was used to perform meta‐analysis on QTLs present in each linkage group separately (Goffinet & Gerber, 2000; Veyrieras et al., 2007). In the first step, QTLs present in each linkage group were clustered together, and the best QTL model was chosen when the lowest criterion values were obtained in at least three of the five selection models (viz., Akaike information criterion [AIC], corrected AIC, AIC model‐3, Bayesian information criterion, and the average weight of evidence criterion). The number of MQTLs on each linkage group was determined in the second step using a chosen model. Finally, variances of initial QTL positions and their intervals were used to calculate the consensus positions and CI (95%) of the meta‐QTLs, respectively (Sosnowski et al., 2012).

### 2.4 Identifying the physical coordinates of the MQTLs

Initially, in order to ascertain the physical coordinates of the MQTLs, the nucleotide sequences of the markers flanking the MQTLs were BLASTed against the pigeonpea reference genome (*C. cajan* acc. Asha v1.0) available at either of the following databases—(i) PulseDB (https://www.pulsedb.org), (ii) Legume Information System (LIS; https://legacy.legumeinfo.org), and (iii) National Center for Biotechnology Information (NCBI; https://www.ncbi.nlm.nih.gov). However, for most of the flanking markers, we did not get the accurate BLAST hits on the concerned linkage groups. Therefore, the genetic positions of markers flanking the MQTLs were converted into physical positions (bp) by using the following conversion factor for each of the linkage groups (Prakash et al., 2022):

$$\begin{array}{ccc} & & \mathrm{Physical}\;\mathrm{position}\;\mathrm{of}\;\text{the}\;m\text{arker}\;(\mathrm{bp})\\ & & =\frac{\mathrm{physical}\;\mathrm{length}\;\mathrm{of}\;\mathrm{chromosome}\;(\mathrm{bp})}{\mathrm{genetic}\;\mathrm{length}\;\mathrm{of}\;\mathrm{thechromosome}\;(\mathrm{cM})}\\ & & \times \;\mathrm{genetic}\;\mathrm{position}\;\mathrm{of}\;\mathrm{the}\;\mathrm{marker}\;(\mathrm{cM}) \end{array}$$

where the physical positions of the linkage groups were obtained from the pigeonpea reference genome available in the PulseDB, whereas the genetic positions of the linkage groups were obtained from the consensus genetic map generated in the present study.

Once the physical coordinates of the MQTLs were obtained, their peak physical positions were calculated as

$$\begin{array}{ccc} & & \mathrm{Peak}\;\mathrm{posi}\mathrm{tion}\;(\mathrm{bp})\;=\mathrm{start}\;\mathrm{posi}\mathrm{tion}\;(\mathrm{bp})\\ & & +\;\frac{\left[\mathrm{end}\;\mathrm{position}\;(\mathrm{bp})\;-\;\mathrm{start}\;\mathrm{position}\;(\mathrm{bp})\right]}{\left[\mathrm{end}\;\mathrm{position}\;(\mathrm{cM})\;-\;\mathrm{start}\;\mathrm{position}\;(\mathrm{cM})\right]}\;\times \;\frac{\text{CI}\;(95\%)}{2} \end{array}$$

### 2.5 Candidate gene (CG) mining within the MQTL regions

In order to extract the information on gene models from the MQTL regions, MQTLs having ≥5 initial QTLs and with less than 5 cM CI (95%) were selected. The gene models and their nucleotide sequences (in FASTA format) were downloaded from the 2 Mb genomic regions (i.e., 1 Mb on each side of the peak position) of the MQTLs (as suggested by Gudi, Saini, et al., 2022; Saini, Srivastava, et al., 2022) by using gene search tool available in the PulseDB (https://www.pulsedb.org/find/genes). These nucleotide sequences were then used for InterPro analysis using the “Transcript Annotation” tool available in the LIS (https://legacy.legumeinfo.org/annot).

### 2.6 Comparison of MQTLs with GWAS‐based MTAs

Data on the most stable and significant MTAs related to the traits under study was collected from three GWA studies published during 2017–2020. These GWA studies involved following three panels—(i) 292 *Cajanus* accessions comprising breeding lines, landraces, and wild species (Varshney et al., 2017), (ii) the association panel involving 89 diverse pigeonpea genotypes (Patil et al., 2017), and (iii) 89 pigeonpea accessions (Zhao et al., 2020). The physical coordinates of the MTAs collected from the above studies were compared with the physical coordinates of the MQTL regions identified during this study to ascertain their co‐localization.

### 2.7 Conserved genomic regions among pigeonpea, chickpea, pea, cowpea, and French bean

Genome synteny and collinearity analyses were conducted to investigate the conserved regions among pigeonpea MQTL regions (using *C. cajan* Asha genome v1.0) and other legume crops (viz., chickpea, *Cicer arietinum* CDC Frontier genome v1.0; pea, *Pisum sativum* Cameor genome v1a; cowpea, *Vigna unguiculata* L. Walp IT97K‐499‐35 genome v1.1; and French bean, *Phaseolus vulgaris* G19833 genome v2.1). The following steps are included in this analysis: (i) the “Tripal Synteny Viewer” available in PulseDB was utilized for the detection of conserved syntenic blocks among the different legumes considered in the present study; (ii) physical coordinates of the MQTLs were compared with those of the conserved synteny blocks to identify the synteny blocks overlapping with the genomic regions of MQTLs; (iii) orthologous gene models available in these synteny blocks were extracted from PulseDB; (iv) physical positions of orthologous gene models were obtained from the “Gene and Transcript Search” option built in PulseDB; (v) physical positions of orthologous gene models were then compared with the physical coordinates of MQTLs. Any MQTL that contained the orthologous gene models for at least one species used for comparison was referred to as an ortho‐MQTL.

## 3 RESULTS

### 3.1 Characterization of QTL studies and collected QTLs

**FIGURE 1:**
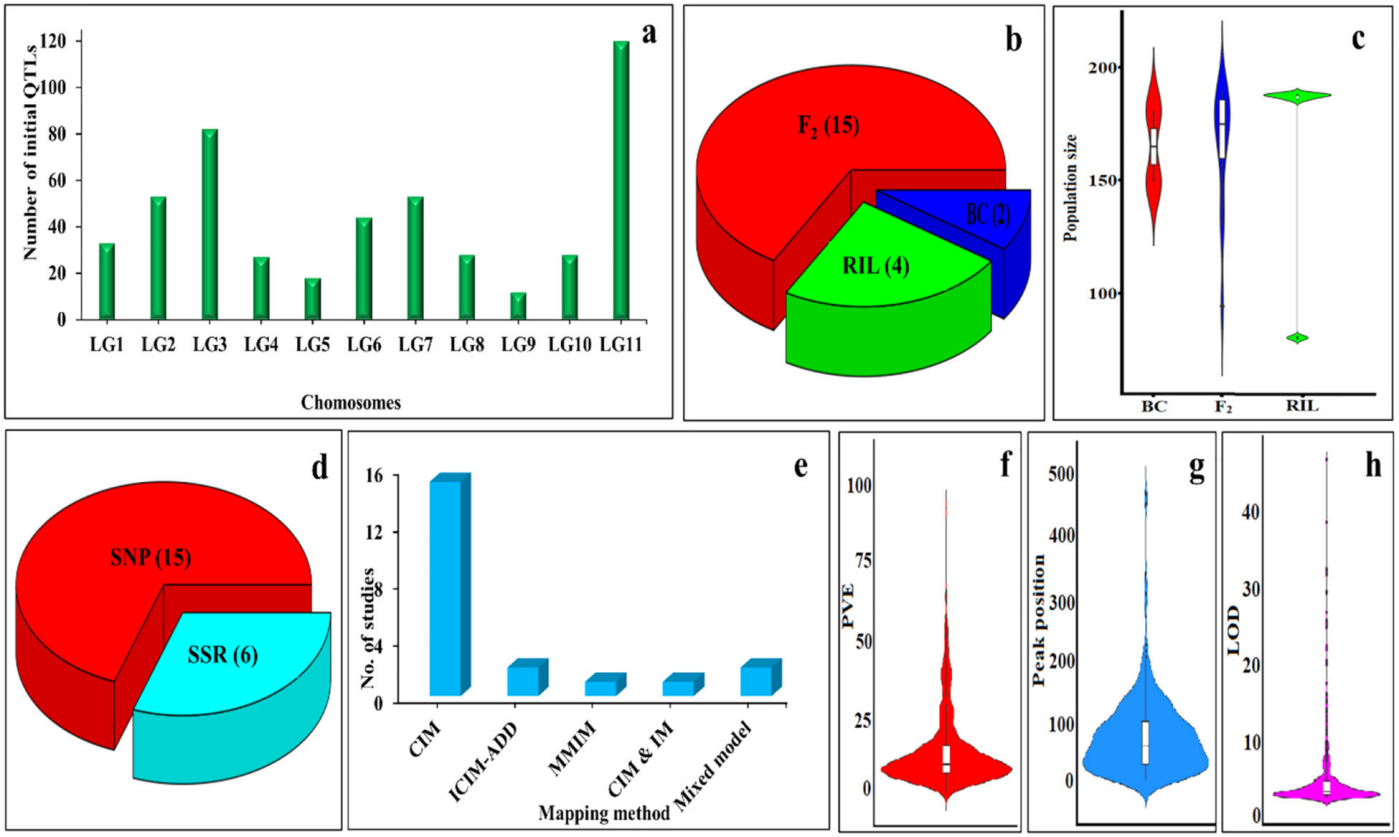
Characteristic features of the initial quantitative trait loci (QTLs): (a) initial number of QTLs available from different linkage groups; (b) number of different types (F_2_, backcross [BC], and recombinant inbred line [RIL]) of populations; (c) size of the different type of mapping populations; (d) number of populations utilizing different types of markers; (e) number of populations utilizing different mapping methods; (f) phenotypic variation explained (PVE) of the QTLs; (g) peak positions of the QTLs; (h) LOD scores of the QTLs utilized.

The QTL information on 498 QTLs associated with traits under study was collected from the 9 mapping studies involving 21 different biparental populations. These QTLs were distributed unequally on different pigeonpea linkage groups, with the maximum number being on LG11 (120 QTLs) and the minimum on LG9 (12 QTLs) (Figure 1a). Among the 21 populations, 4 were RIL populations, 15 were F_2_ populations (including one F_2:3_), and 2 were BC populations, with population sizes ranging from 80 to 188, 94 to 190, and 149 to 181, respectively (Figure 1b,c; Tables S1 and S2). Mapping populations that used different types of markers and QTL mapping methods are presented in Figure 1d,e, respectively. The majority of the interval mapping studies employed SNPs as markers (15 mapping populations) and composite interval mapping as a mapping approach (15 mapping populations). Among the studied traits, agronomic traits (398 QTLs) and fertility restoration (8 QTLs) had the highest and lowest number of QTLs, respectively. However, the number of QTLs associated with disease resistance and seed quality traits was 35 and 57, respectively. Of the total QTLs, 307 QTLs were available with the PVE values. Among them, 155 QTLs had a PVE value of less than 10%. The PVE values of each QTL varied from 0.7% to 91.3%, with a mean value of approximately 14% (Figure 1f). Figure 1g shows that the majority of the initial QTLs had genetic peaks ranging from 0 to 100 cM. Furthermore, the LOD scores of individual QTLs varied from 2.5 to 46.2, with 259 QTLs having LOD scores of less than 5 (Figure 1h).

### 3.2 Pigeonpea consensus map

**FIGURE 2:**
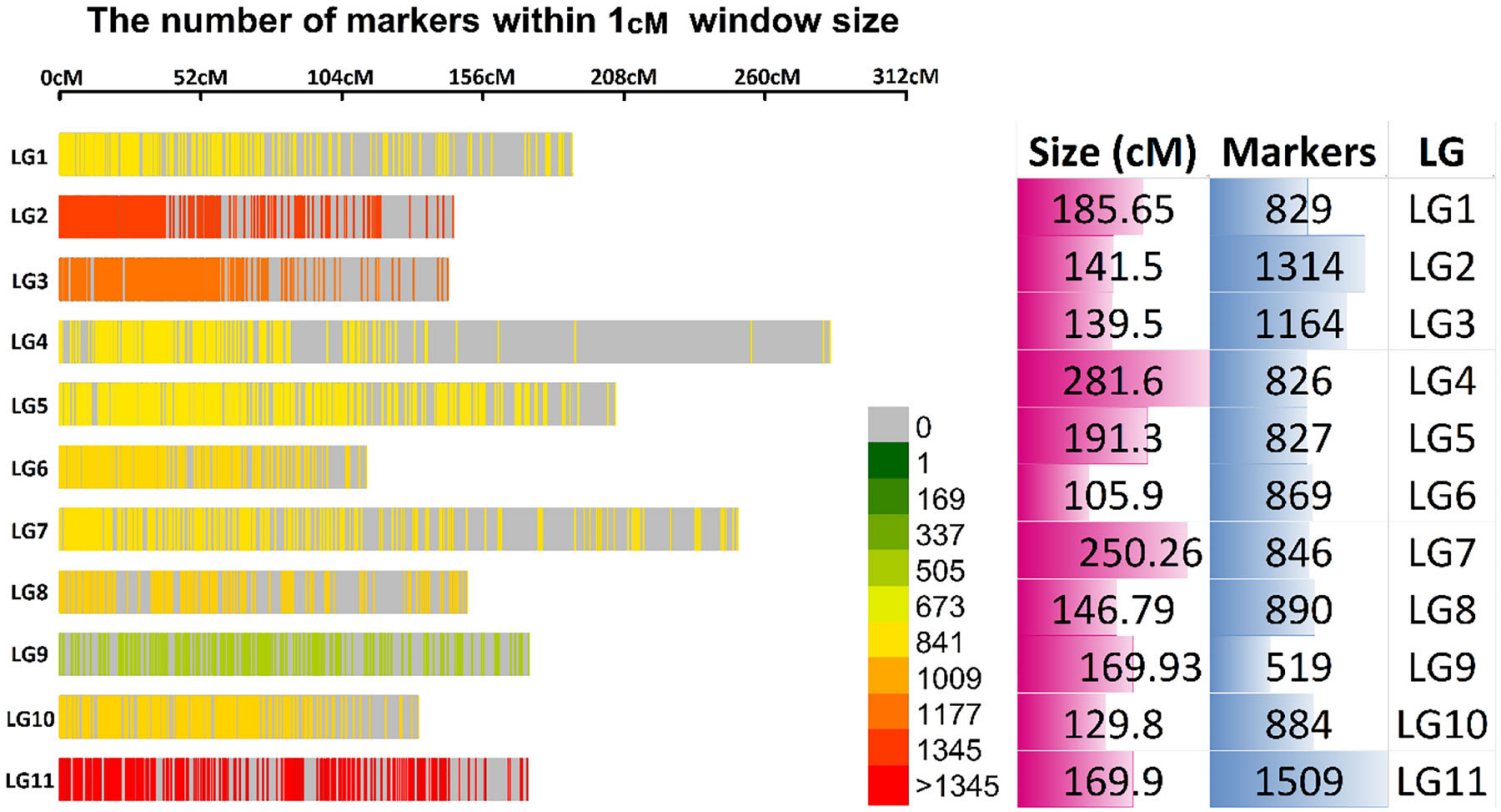
“PigeonPea_ConsensusMap_2022” showing the marker density, the length of the linkage group (cM) (pink column), and the number of markers in each linkage group (blue column).

A consensus genetic map, termed “PigeonPea_ConsensusMap_2022,” was constructed, which initially had 10,522 markers and covered a genetic length of 2819.89 cM. The total number of markers was reduced from 10,522 to 10,477 by removing a few that showed significant gaps at the ends of the chromosomal maps while taking into account the peak genetic positions of the initial QTLs. This exercise caused the total genetic length to be reduced to 1912.13 cM. The individual linkage groups in the consensus map showed substantial variation in their genetic lengths, with LG6 (105.9 cM) being the smallest and LG4 (281.6 cM) being the largest linkage groups, respectively, and also for the number of markers mapped on individual linkage groups, with the maximum number being on LG11 (1509) and the minimum on LG9 (519), respectively (Figure 2). The marker density (markers/cM) on each of the linkage groups varied from 2.93 (LG4) to 9.29 (LG2), respectively, with an average of 5.98 markers/cM on the whole genome. Furthermore, two ends of the individual linkage groups showed substantial variations in marker density, which is owing to the use of different linkage maps with differing numbers and types of markers for the construction of consensus map (Figure 2; Table S4).

### 3.3 QTL projection and MQTL analysis

**FIGURE 3:**
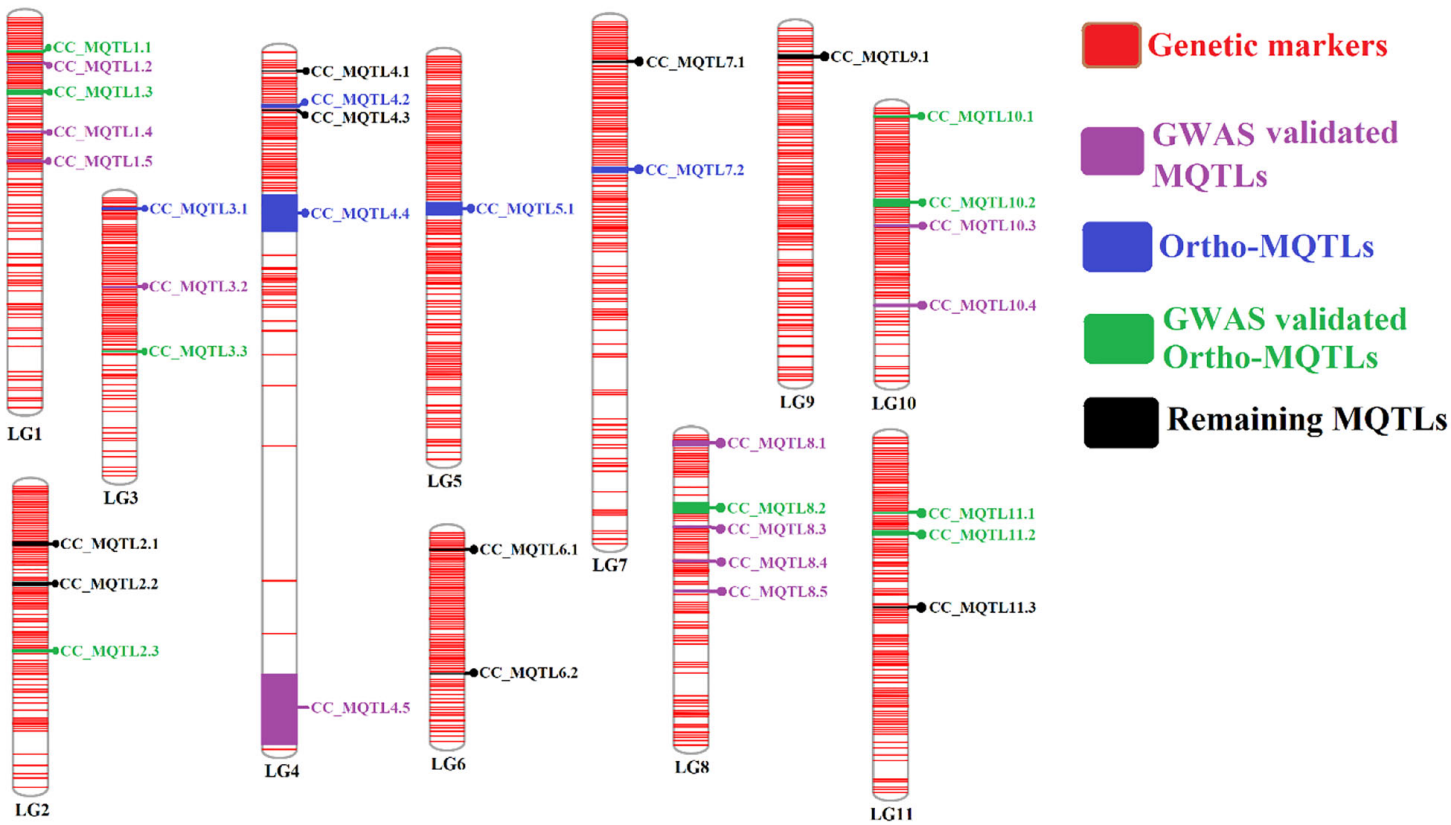
The distribution of identified meta‐quantitative trait loci (MQTLs) on different linkage groups. The explanation for the various colors used to depict the MQTLs is provided at the right side of the figure.

Of the total 498 QTLs, 307 QTLs having the necessary information on PVE values and genetic positions of the flanking markers were chosen for the projection on PigeonPea_ConsensusMap_2022. The QTLs (191 QTLs) lacking necessary information were excluded from the further analysis. Only 203 (66.12%) of the total 307 QTLs could be projected on the consensus map. A total of 104 QTLs could not be projected because of either of the following reasons: (i) lack of shared markers between consensus and genetic maps; (ii) inclusion of initial QTLs with larger CI. Furthermore, it was observed that, of the 203 projected QTLs, 186 QTLs could be clustered into 34 MQTLs, whereas 17 QTLs remained as singletons (Table 1; Figure 3).

**TABLE 1:** Summary of pigeonpea meta‐quantitative trait loci (MQTLs) associated with different traits

MQTLs	Genetic position (in cM)	Flanking markers	Physical position (in bp)	No. of projected QTLs	Associated trait
CC_MQTL1.1	16.98–17.85	Affx_123334476—A_SNP_0124	826,682.5–869,051.5	10	Agronomic and seed quality traits
CC_MQTL1.2	22.35–22.65	A_SNP_1534—CcM02891	1088,445–1103,055	2	Agronomic traits
CC_MQTL1.3	35.86–37.09	Affx_123347907—Affx_123305310	1746,139–1806,040	2	Agronomic and seed quality traits
CC_MQTL1.4	55.25–55.97	Bin1.06—A_SNP_0512	2690,675–2725,739	2	Agronomic traits
CC_MQTL1.5	69.19–70.29	HA_SSR0194—A_SNP_0720	3369,553–3423,123	4	Agronomic traits and disease resistance
CC_MQTL2.1	27.48–29.08	A_SNP_0092—S1_345426948	3517,440–3722,240	8	Agronomic traits, disease resistance, seed quality traits
CC_MQTL2.2	46.96–48.43	CcM0399—AHSSR201	6010,240–6198,400	2	Seed quality traits
CC_MQTL2.3	79.8–80.27	S2_6037523—S2_5078598	10,213,760–10,273,920	6	Agronomic and seed quality traits
CC_MQTL3.1	5.92–6.48	S3_21244595—S3_10282824	964,960–1056,240	27	Agronomic traits, disease resistance, seed quality traits
CC_MQTL3.2	43.38–43.48	CcM01453—DRDRP_144_1165	7070,940–7087,240	30	Agronomic and seed quality traits
CC_MQTL3.3	74.93–74.96	Affx_123323773—S3_14273504	12,212,775–12,217,665	7	Agronomic and seed quality traits
CC_MQTL4.1	9.12–9.24	CcM01962—Affx_123350329	243,504–246,708	4	Agronomic traits and disease resistance
CC_MQTL4.2	26.86–27.09	Affx_123324362—S1_157985149	717,028.5–723,169.5	2	Agronomic and seed quality traits
CC_MQTL4.3	28.04–28.6	Affx_123315322—S1_186395748	748,668–763,620	7	Agronomic and seed quality traits
CC_MQTL4.4	69.35–87.19	CcM00824—CcGM16612	1851,645–2327,973	3	Agronomic and seed quality traits
CC_MQTL4.5	301.34–335.43	AHSSR231—AHSSR251	8045,645–8955,848	3	Agronomic and seed quality traits
CC_MQTL5.1	71.18–77.43	AX_165402747—SCP_11093_644	1359,443–1478,818	7	Agronomic traits
CC_MQTL6.1	8.28–9.13	Affx_123342177—Affx_123323527	1696,375–1870,625	5	Agronomic traits and fertility restoration
CC_MQTL6.2	68.13–68.37	S6_14282225—A_SNP_1764	13,966,650–14,015,850	5	Agronomic and seed quality traits
CC_MQTL7.1	19.15–20.6	CcM02176—SCP_13976_143	938,105–1009,155	3	Agronomic traits and disease resistance
CC_MQTL7.2	70.16–73.15	SCP_4746_1719—CcM00752	3437,595–3584,105	2	Agronomic traits
CC_MQTL8.1	3.85–6.02	S1_041628935—S1_287205977	465,245–727,815	3	Agronomic traits
CC_MQTL8.2	33.81–38.21	CcM02004—CcM0413	4091,010–4623,410	2	Agronomic traits
CC_MQTL8.3	44.04–45.85	CcM01282—CcM02911	5328,235–5547,245	3	Agronomic and seed quality traits
CC_MQTL8.4	62–62.93	Affx_123301653—Affx_123301298	7501,395–7613,925	2	Agronomic and seed quality traits
CC_MQTL8.5	75.37–75.4	CcGM17620—Affx_123314293	9119,165–9122,795	2	Agronomic traits and disease resistance
CC_MQTL9.1	13.13–15.06	CcM00588—CcM02781	640,500–734,684	3	Agronomic traits and disease resistance
CC_MQTL10.1	4.52–5.52	Affx_123331803—A_SNP_0268	655,400–800,400	2	Agronomic and seed quality traits
CC_MQTL10.2	44.74–48.3	CcM00673—DRDRP_741_735	6487,300–7003,500	2	Agronomic traits
CC_MQTL10.3	57.73–58.4	CcM00268—CSCSP_3040_1024	8370,125–8467,275	2	Agronomic traits
CC_MQTL10.4	95.16–96.25	Bin10.10—A_SSR_0408	13,797,475–13,955,525	2	Agronomic traits
CC_MQTL11.1	36.08–36.85	S1_186504993—S11_8867457	8694,075–8879,645	8	Agronomic traits, disease resistance, seed quality traits
CC_MQTL11.2	45.97–47.47	S11_36998432—Affx_123318683	11,078,770–11,440,270	3	Agronomic and seed quality traits
CC_MQTL11.3	82.3–82.34	S11_39257442—S11_29670540	19,834,300–19,843,940	11	Agronomic and seed quality traits

The number of MQTLs on each linkage group varied from one (each on LG5 and LG9) to five (LG1, LG4, and LG8) (Figure S1a; Table S5). Among the 34 MQTLs, 33, 1, 8, and 19 MQTLs contained initial QTLs for agronomic traits, fertility restoration, disease resistance, and seed quality traits, respectively (Figure S1b). The average CI of MQTLs (i.e., 2.54 cM) was 3.37 times lower than the CI of initial QTLs (i.e., 8.56 cM), and there were substantial differences among the different linkage groups (Figure S1c). The MQTLs also varied for the initial number of QTLs, which ranged from 2 in several MQTLs to a maximum of 30 QTLs in CC_MQTL3.2, with 12 MQTLs involving a minimum of 5 initial QTLs (Figure S1d). Three of these 12 MQTLs were located on LG3 (viz., CC_MQTL3.1, CC_MQTL3.2, and CC_MQTL3.3), with the remaining 9 MQTLs located on the following linkage groups: LG1 (1), LG2 (2), LG4 (1), LG5 (1), LG6 (2), and LG11 (2). Furthermore, 10 MQTLs were found to be solely associated with single trait, whereas 21 and 3 MQTLs were shown to be associated with 2 and 3 traits, respectively (Figure S1e; Table 1). However, the MQTLs, including initial QTLs for all four studied traits, were not identified.

### 3.4 Candidate genes available from the MQTL regions

A total of 2255 gene models were extracted from the 2 Mb regions of 12 MQTLs (having ≥5 initial QTLs with ≤5 cM CI) (Table S6). The individual MQTLs varied for the number of gene models, with the maximum genes available from CC_MQTL3.3 (220 gene models) and the minimum genes available from CC_MQTL2.1 (155 gene models) (Table S7). The identified MQTL regions also had a cluster of genes associated with some specific superfamilies, such as (i) glycoside hydrolase superfamily, (ii) protein kinase‐like domain superfamily, (iii) leucine‐rich repeat domain superfamily, (iv) glucanase domain superfamily, and (iv) ribonuclease H‐like superfamily (Table S6). Furthermore, the gene ontology (GO) analysis identified a number of important GO terms. Some of them include (i) *molecular functions*: protein dimerization, *O*‐methyltransferase, oxidoreductase, protein phosphatase, ubiquitin–protein transferase, protein kinase, nucleic acid binding, Zn binding, protein binding, and ATP binding; (ii) *biological processes*: transcription regulation, protein ubiquitination, protein phosphorylation, DNA integration, signal transduction, transmembrane amino acid transport, DNA repair, and so on; (iii) *cellular components*: mediator complex, protein phosphatase type 2A complex, intracellular anatomical structure, signal peptidase complex, an integral component of cell membrane, and so on (Table S6).

Further, the “InterPro description” enabled the identification of a large number of gene models associated with agronomic traits (154), fertility restoration (36), disease resistance (53), and seed quality traits (7) within the different MQTL regions (Figure S2). Based on the available literature, these genes are believed to be responsible for plant growth and development, flower initiation, biotic stress tolerance, seed development, and seed storage protein synthesis. Among them, 105 gene models (called high‐confidence CGs) were available from some trait‐specific MQTLs (Table S8). For instance, of the total 36 genes responsible for fertility restoration, only 1 gene, *gene‐KK1_011505*, was considered a high‐confidence CG, as it was located on the MQTL (i.e., CC_MQTL6.1) carrying initial QTLs for this trait. However, the remaining 35 genes were identified from the MQTLs with no initial QTLs for fertility restoration and were not considered high‐confidence CGs. Similarly, the high‐confidence CGs associated with each of these traits are presented in Table S8.

### 3.5 Comparison of MQTLs with GWAS‐based MTAs

The physical positions of pigeonpea MQTL regions identified in the present study were compared with the 197 MTAs reported from previous GWAS (Table S9). Among the 34 MQTLs, as many as 8 MQTLs (23.53%) were found to be co‐localized with at least 1 MTA (Figure 3; Table S9). Among the eight GWAS verified MQTLs, two MQTLs (CC_MQTL1.5 and CC_MQTL3.3) were co‐localized with only one MTA, three MQTLs (CC_MQTL4.5, CC_MQTL11.1, and CC_MQTL11.2) were co‐localized with two MTAs, two MQTLs (CC_MQTL2.3 and CC_MQTL3.2) were co‐localized with three MTAs, and only one MQTL (CC_MQTL8.5) was co‐localized with four MTAs. Furthermore, among the 21 studied traits, MQTLs associated with only 6 traits (Fusarium wilt, plant height, days to flowering, pods per plant, seed weight, and seed yield) were reported to be overlapped with GWAS‐MTAs, whereas MQTLs associated with the remaining traits were not, which may be owing to the availability of a very small number of GWAS‐MTAs for the particular trait.

### 3.6 Conserved genomic regions between pigeonpea and other legumes

**FIGURE 4:**
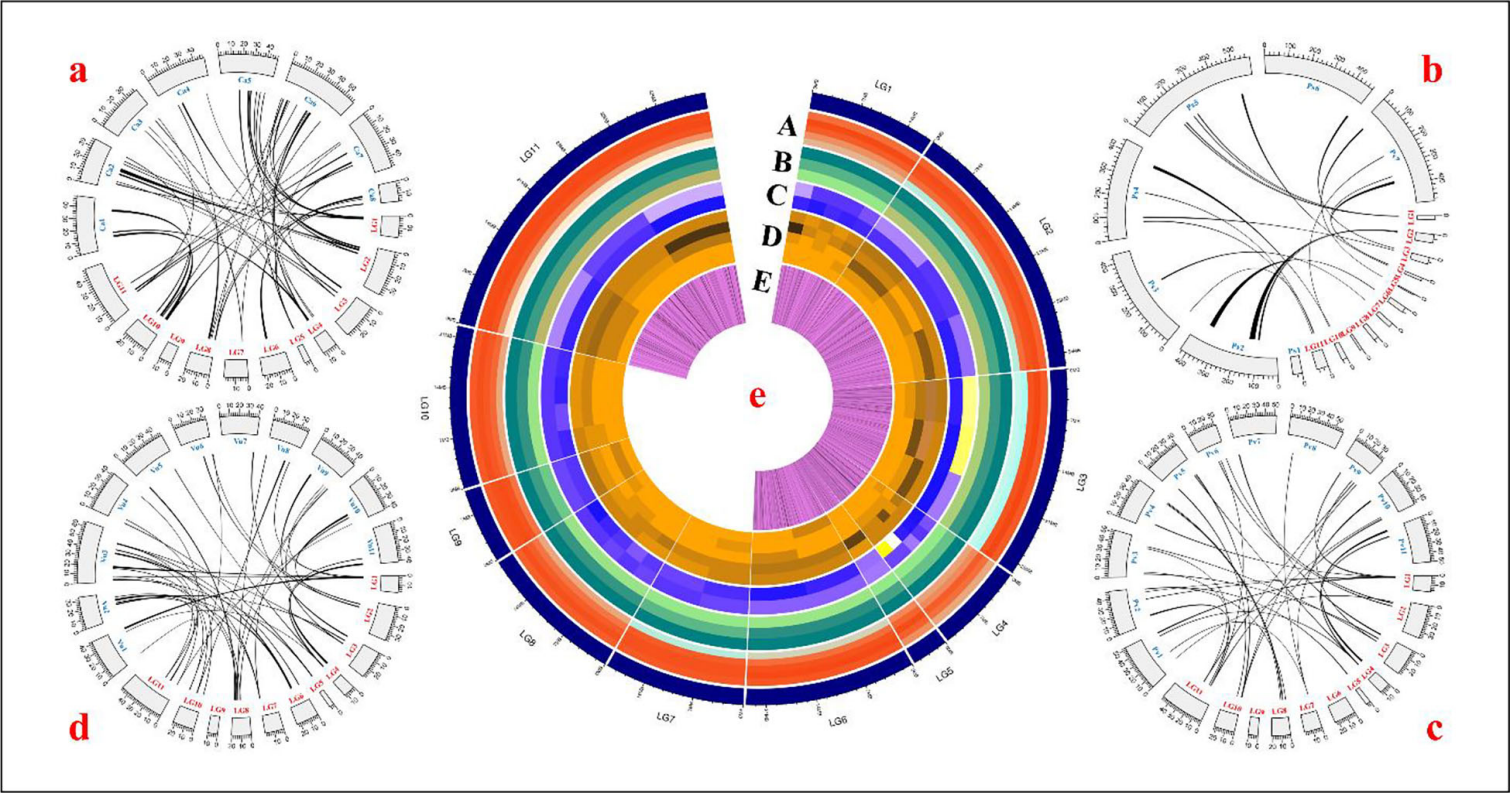
Circular diagrams depicting the conserved synteny blocks and different features of quantitative trait loci (QTLs) and meta‐quantitative trait loci (MQTLs): (a) the conserved synteny blocks of MQTL regions with chickpea (*Cicer arietinum*; Ca); (b) the conserved synteny blocks of MQTL regions with pea (*Pisum sativum*; Ps); (c) the conserved synteny blocks of MQTL regions with cowpea (*Vigna unguiculata*; Vu); (d) the conserved synteny blocks of MQTL regions with French bean (*Phaseolus vulgaris*; Pv); (e) sub‐tracks (from outside to inside) in **Circle A** depicts the linkage group (LG) wise distribution of the initial number of QTLs, projected QTLs on consensus map, number of MQTLs, average confidence interval (CI) of initial QTLs, and average CI of MQTLs; in **Circle B** depicts the number of markers, length of the linkage groups, and marker density in the PigeonPea_ConsensusMap_2022; in **Circle C** depicts the number of projected QTLs and average CI of individual MQTLs; in **Circle D** depicts the initial number of QTLs for fertility restoration, disease resistance, seed quality traits, agronomic traits in each MQTL and the number of traits in each MQTLs; and **Circle E,** Gene density distribution in the MQTL regions. The outer circle represents pigeonpea linkage groups (LG1–LG11).

Synteny analysis uncovered 3060 conserved syntenic blocks of the pigeonpea genome with chickpea (783), pea (642), cowpea (808), and French bean (827). Of the total 3060, 113 synteny blocks overlapping with the genomic regions for only 26 MQTLs were identified. Furthermore, among the 113 synteny blocks, 26 synteny blocks were overlapped with 19 MQTLs in chickpea (Figure 4a), 24 synteny blocks overlapped with 17 MQTLs in pea (Figure 4b), 35 synteny blocks overlapped with 20 MQTLs in cowpea (Figure 4c), and 28 synteny blocks overlapped with 18 MQTLs in French bean (Figure 4d; Table S10). The conserved synteny blocks overlapping with the following 11 MQTLs‐CC_MQTL1.1, CC_MQTL1.3, CC_MQTL2.2, CC_MQTL2.3, CC_MQTL3.3, CC_MQTL4.2, CC_MQTL4.3, CC_MQTL5.1, CC_MQTL10.1, CC_MQTL11.1, and CC_MQTL11.3 were identified in all the four legumes. Furthermore, the synteny blocks overlapping with five MQTLs (viz., CC_MQTL2.1, CC_MQTL4.4, CC_MQTL8.5, CC_MQTL10.3, and CC_MQTL10.4) were unique. Of these five MQTLs, two (CC_MQTL2.1 and CC_MQTL10.3) were unique to chickpea, two (CC_MQTL4.4 and CC_MQTL10.4) were unique to French bean, and one (CC_MQTL8.5) was unique to cowpea.

Of the 113 synteny blocks, 67 synteny blocks overlapped with 20 MQTLs (from now onward called ortho‐MQTLs) yielded 117 pigeonpea genes, which were orthologous to 265 genes in the 4 legume crops (Table S11). The remaining synteny blocks did not give orthologous genes of pigeonpea (from the MQTL regions) in any of the legume crops studied. The highest number of orthologous genes (i.e., 90 genes) was obtained in cowpea (from 16 ortho‐MQTL regions) and French bean (from 14 ortho‐MQTL regions) for 72 and 80 pigeonpea genes, respectively. Further, the least number of orthologous genes (i.e., 37 genes) was obtained in pea (from nine ortho‐MQTL regions) for 33 pigeonpea genes. In addition, 48 orthologous genes of 42 pigeonpea genes were identified from 10 ortho‐MQTL regions in chickpea. Furthermore, among these 117 genes, 9 orthologous genes obtained from 4 ortho‐MQTL regions (viz., CC_MQTL2.2, CC_MQTL5.1, CC_MQTL10.1, and CC_MQTL11.1) were conserved in all the 4 legume crops. However, 50 orthologous genes obtained from the 5 ortho‐MQTL regions (viz., CC_MQTL1.5, CC_MQTL2.1, CC_MQTL4.3, CC_MQTL4.4, and CC_MQTL8.4) were unique to any of the four crops investigated.

## 4 DISCUSSION

Molecular markers are considered the potential tools for genomics research and molecular breeding for genetic improvement in any crop plant. Compared to other legume crops, molecular breeding for genetic enhancement of economically important traits is still in infancy in pigeonpea. However, recent initiatives have been undertaken to improve the major traits using novel genomic tools, including high‐throughput genotyping and next‐generation sequencing (Varshney et al., 2012). Such studies have resulted in the development of high‐density linkage and physical maps in pigeonpea (e.g., Varshney et al., 2013).

In pigeonpea, various types of molecular markers have been developed, which have led to the identification of a number of QTLs affecting important agricultural traits. Transferring these genes/QTLs into elite genetic backgrounds may speed up the varietal development process and also improve the targeted trait significantly (Bohra et al., 2020; Singh et al., 2022). Successful utilization of QTLs in molecular cloning and marker‐assisted breeding (MAB) depends on their effect sizes and consistency across the multiple environments and genetic backgrounds. However, most of the QTLs are minor QTLs with low PVE values (less than 10%) and a large CI, making them less useful in MAB (Gudi, Saini, et al., 2022). Furthermore, QTLs identified in one mapping population might not be useful in a breeding effort involving a different population (Yang et al., 2021). The shortcomings of these QTLs encouraged us to integrate QTL results from various studies to provide useful information on robust and stable QTLs or MQTLs associated with the important quantitative traits, so that the scientific community could make better use of them.

The MQTL analysis has been considered the most important strategy for integrating QTLs in order to overcome heterogeneity present in different studies (Goffinet & Gerber, 2000). It has a great capability of identifying stable and reliable MQTLs by compiling information from several mapping studies involving diverse genetic backgrounds and multiple environments (Chardon et al., 2004; Kumar et al., 2021; Pal et al., 2022, 2021; Saini et al., 2021; Saini, Chahal, et al., 2022; Saini, Srivastava, et al., 2022; Welcker et al., 2011; Gudi, Saini, et al., 2022).

Meta‐analysis has been performed for a number of traits in a wide range of species, including wheat, rice, maize, sorghum, barley, and tomato (Aquib & Nafis, 2022; Ayenan et al., 2018; Kaur et al., 2021; Saini, Srivastava, et al., 2022; Sandhu et al., 2021; Zhang et al., 2017). However, there are no studies available on the meta‐analysis of QTLs in pigeonpea. Therefore, this is the first as well as most comprehensive study in pigeonpea where a meta‐analysis of the available QTLs identified 34 MQTLs associated with agronomic traits, fertility restoration, disease resistance, and seed quality traits. Additionally, because of the accessibility of NGS‐based high‐throughput marker systems, advancements in QTL mapping methodologies, and a rise in geneticists’ interest in genetic dissection studies, linkage‐based QTL mapping studies have recently become widespread. Just to exemplify, during the final writing of this paper, three linkage‐based QTL mapping studies were published (Saxena, Kale, et al., 2020; Saxena, Molla, et al., 2020; Singh, Sinha, et al., 2022), which reported the identification of several QTLs for fertility restoration, days to flowering, and yield‐related traits. Comparison of the physical positions of these QTLs with the physical coordinates of MQTLs revealed the co‐localization of five of these QTLs with three MQTLs (viz., CC_MQTL7.2, CC_MQTL8.2, and CC_MQTL8.4) identified during the present study.

MQTLs involving a large number of initial QTLs are of foremost importance from the breeding point of view. Such MQTLs involving as many as 30 initial QTLs (i.e., CC_MQTL3.2) were identified in the present study, which is far greater than some of the previous reports in other legumes (Vasconcellos et al., 2017). Furthermore, QTL information included from diverse studies also enabled the reduction in CI of QTLs and also helped in improving the precision of CG mining from the significant MQTL regions. The average CI of MQTLs was 3.37 times lower than the CI of the initial QTLs used in this study. Of the 34 MQTLs, 12 high‐confidence MQTLs with a smaller CI (≤5 cM) and a greater number of initial QTLs (≥5) were identified. Two of these high‐confidence MQTLs, CC_MQTL5.1 and CC_MQTL6.1, were associated with agronomic traits and fertility restoration, respectively. Furthermore, seven of these high‐confidence MQTLs (CC_MQTL1.1, CC_MQTL2.3, CC_MQTL3.2, CC_MQTL3.3, CC_MQTL4.3, CC_MQTL6.2, and CC_MQTL11.3) can be used for the simultaneous improvement of different agronomic and quality traits. However, the remaining three MQTLs (CC_MQTL2.1, CC_MQTL3.1, and CC_MQTL11.1) were associated with agronomic traits, disease resistance, and seed quality traits, which may facilitate the simultaneous improvement of all these traits during pigeonpea breeding.

The 12 high‐confidence MQTLs revealed in this work might pave the way for the positional cloning and functional characterization of genes associated with studied traits in pigeonpea. Furthermore, these might pave the way for marker‐aided breeding between populations, adding to pigeonpea genetic improvement. To our knowledge, there are no reports on GS for any of the traits in pigeonpea. Therefore, by using markers (either peak or flanking markers) associated with these MQTLs as fixed effects in the different GS models, one can effectively conduct the GS in pigeonpea as this may improve the efficiency of prediction models in GS.

### 4.1 Candidate genes identified within the MQTL regions

The information on the physical positions of the MQTLs is required to extract the CGs. However, the nucleotide sequences of most of the markers either provided poor hits (low identity percentages and scores, and also high *E* values) or provided hits on chromosomes other than the one intended to be targeted. Therefore, physical anchoring of the MQTLs on the pigeonpea reference genome by BLAST searches could not be performed. For this reason, we retrieved the physical coordinates of MQTLs by using a conversion factor involving physical and genetic lengths of linkage groups and genetic positions of markers flanking the MQTLs, as reported in one earlier report of meta‐analysis (Prakash et al., 2022). MQTLs are thought to be the potential target for identifying CGs associated with the traits under consideration. Earlier studies conducted in various cereals and legume crops revealed that the MQTL regions as gene‐rich regions of the genome (Quraishi et al., 2017; Yang et al., 2021). For instance, 26 CGs associated with leaf spot resistance (Lu et al., 2018), 161 CGs associated with plant height (Lv et al., 2018), and 686 CGs associated with seed number (Chen et al., 2019) were identified in groundnut. Furthermore, 10,137 amino acid sequences were predicted from the MQTL regions in soybean (Chen et al., 2021). In the present study, CG mining within 2 Mb regions of 12 MQTLs identified 2255 gene models; at least some of these genes should be associated with the studied traits. Based on InterPro description, GO analysis, and a thorough survey of the available literature, 105 high‐confidence CGs (out of 2255 gene models) associated with all studied traits were identified and recommended for future genetic and basic studies.

These CGs were mainly associated with fertility restoration, response to biotic stresses, regulation of flowering time, flower and seed development, transport and accumulation of seed storage proteins, and plant growth and development. Of the 105 high‐confidence CGs, 92 CGs associated with important agronomic traits were identified from 11 MQTL regions. These CGs were found to encode important proteins associated with (i) flower initiation and development (CCT domain, FHY3/FAR1, glutaredoxin, WD40 repeat domain, AP2 domain, Frigida‐like, and K‐homology domain); (ii) seed growth and development (B3 DNA binding domain, Dof‐type, and F‐box associated interaction domain); and (iii) plant growth and development (UDP‐glucuronosyl/UDP‐glucosyltransferase, protein phosphatase 2C family, basic helix‐loop‐helix domain, Leucine‐rich repeat domain superfamily, F‐box domain, etc.) (Figure S2; Table S8).

The glutaredoxin, WD40, AP2, Frigida‐like genes, *FLK*, and *FLC* genes are involved in floral development through petal and flower primordia formation (Hofmann, 2009). The basic helix–loop–helix, UDP‐glucosyltransferase protein families, leucine‐rich repeat superfamily, AUX/IAA domain, and GRAS transcription factors play an important role in regulating seed germination, root growth, seed maturation, and dormancy (e.g., Hao et al., 2021; Liu et al., 2015). One CG, *gene‐KK1_011505*, located on CC_MQTL6.1, encodes the pentatricopeptide repeat proteins, which interfere with mitochondrial RNA processing machinery and thus aid in the regulation of fertility restoration (Gudi et al., 2020).

Identifying the durable resistant genes and their transfer into the elite genetic background reduces the economic loss caused by different pathogens. In the present study, we identified the 10 CGs associated with resistance to Fusarium wilt and SMD (Table S8). These genes encode the calmodulin‐binding domain, WRKY domain, Ankyrin repeat, POZ domain, transcription factor TGA‐like domain, and P‐loop containing nucleoside triphosphate hydrolase. The calmodulin‐binding proteins and P‐loop containing nucleoside triphosphate hydrolases contain a number of kinases involved in the disease resistance signaling pathways (Zheng et al., 2004). The WRKY proteins regulate the response to biotic stresses through the modulation of salicylic acid, jasmonic acid, and the ethylene signaling pathways.

Improving the seed quality and nutritional traits in pigeonpea will give added benefits to consumers. The present study identified seven CGs involved in the synthesis and transport of seed storage proteins (Table S8). These genes encode the seed storage helical domain, hydrophobic seed protein domain, RINT‐1/Tip20, and Myc‐type proteins. The RINT‐1/TIP20 controls the transport of seed storage proteins in the developing endosperms (Li et al., 2006, 2013). The Myc‐type of transcription factors regulates seed development (i.e., seed size and weight) and controls the accumulation of seed storage proteins.

### 4.2 Comparison of MQTLs with GWAS results

GWAS has become an increasingly popular and efficient method that permits the genetic dissection of complex traits through high‐resolution QTL mapping (Pang et al., 2020). The MTAs identified in association panels are used to pinpoint CGs underlying QTLs, which in turn accelerates breeding processes via MAB (Varshney et al., 2017). Introducing GWAS in pigeonpea facilitated the identification of MTAs for Fusarium wilt and several other agronomic traits (Table S9) (Patil et al., 2017; Varshney et al., 2017; Zhao et al., 2020). Among them, two studies used the pigeonpea association panel and pangenome to discover the GWAS‐MTAs. In the present study, around 24% (8/34) of the identified MQTLs (two for Fusarium wilt and six for agronomic traits) were observed to be overlapped with MTAs. All reported MQTLs were not found to be overlapped with GWAS_MTAs, as reported in previous findings (Gudi, Saini, et al., 2022; Saini et al., 2021; Saini, Srivastava, et al., 2022) and the current study. This could be because (i) neither MQTL analysis nor the GWAS approach took into account all of the genetic variations available in the gene pool; (ii) the genetic material used in meta‐analysis and GWAS were entirely different; and (iii) GWAS is intended to discover MTAs with a minor allele frequency of more than 5%. However, linkage mapping studies can detect rare alleles with larger phenotypic effects (with a minor allele frequency of ≤5%); and (iv) the number of GWAS‐MTAs used for comparison was very small.

The most stable and consistent MQTL is the one that overlaps with multiple GWAS‐MTAs and includes many QTLs from different interval mapping studies. Three such MQTLs (CC_MQTL2.3, CC_MQTL3.2, and CC_MQTL11.1) having ≥5 initial QTLs with <2 cM CI were identified during the present study. These MQTLs are thought to be promising and could be used in marker‐assisted trait improvement in pigeonpea.

### 4.3 Conserved genomic regions of pigeonpea MQTLs in other legume crops

Identifying the conserved genomic regions of MQTLs in other legume crops may increase their utility. Considering high synteny among the legume crops (Gujaria‐Verma et al., 2014), the pigeonpea MQTLs identified in the present study were used to discover the conserved synteny blocks in four legume crops (viz., chickpea, pea, cowpea, and French bean). The ortho‐MQTLs and the orthologous genes discovered in this study might be used in future to develop conserved orthologous set markers for legume improvement through marker‐assisted breeding. Furthermore, conserved ortho‐MQTLs and their corresponding orthologous genes may provide an opportunity to study the common evolutionary pathways that led to the establishment of legume lineages.

## 5 CONCLUSIONS

Plant breeders are deeply concerned with developing high‐yielding, stress‐resilient crop varieties with improved nutritional qualities. In the present study, efforts were made to understand the complex genetic architecture of four major trait categories (i.e., agronomic traits, fertility restoration, disease resistance, and seed quality traits) in pigeonpea through MQTL analysis and comparative genomics. The MQTLs and CGs identified in this study may facilitate MAB and trait improvement in pigeonpea. Furthermore, information on markers associated with the MQTLs can be used in the GS models to increase the prediction accuracy for the targeted traits in pigeonpea. The ortho‐MQTL regions and their corresponding orthologous genes identified in this study may facilitate the interspecies transfer of genomic information among the legume crops. Pigeonpea breeders can make better use of the most promising MQTLs (viz., CC_MQTL2.3, CC_MQTL3.2, and CC_MQTL11.1) and CGs identified during the present study for genetic enhancement of traits in question.

## AUTHOR CONTRIBUTIONS

**Priyanka Halladakeri**: Conceptualization, Data curation, Formal analysis, Methodology, Resources, Software, Validation, Visualization, Writing – original draft. **Santosh Gudi**: Conceptualization, Data curation, Formal analysis, Investigation, Methodology, Project administration, Software, Supervision, Visualization, Writing – original draft, Writing – review & editing. **Sabina Akhtar**: Conceptualization, Writing – review & editing. **Gurjeet Singh**: Data curation, Formal analysis, Methodology, Resources, Writing – original draft, Writing – review & editing. **Dinesh Kumar Saini**: Conceptualization, Investigation, Project administration, Visualization, Writing – review & editing. **Harshavardan Hilli**: Data curation, Methodology, Resources, Software, Writing – original draft. **Amar Sakure**: Investigation, Project administration, Visualization, Writing – review & editing. **Sneha Macwana**: Investigation, Project administration, Supervision, Visualization, Writing – review & editing. **Reyazul Rouf Mir**: Investigation, Project administration, Visualization, Writing – review & editing.

## CONFLICT OF INTEREST STATEMENT

The authors of this manuscript declare no conflict of interests.

## Supporting information

Supporting Information 2
Table S1 Summary of the individual QTL mapping studies utilized in the present study.
Table S2 List of identified QTLs from the 9 independent studies involving 21 populations and the related information (QTLs highlighted with yellow color were not been used for projection).
Table S3 Traits grouped into different major trait categories.
Table S4 PigeonPea_ConsensusMap_2022 constructed in the present study.
Table S5 Details on identified MQTLs detected in the present study.
Table S6 Details on gene models identified in the present study.
Table S7 Number of initial QTLs, projected QTLs, and gene models identified in the different linkage groups.
Table S8 High‐confidence candidate genes (CGs) identified during the present study.
Table S9 MQTLs co‐localized with MTAs identified in the earlier GWA studies.
Table S10 Conserved synteny blocks identified between pigeonpea (MQTL regions) and other legume crops (pea, French bean, cowpea, and chickpea).
Table S11 Conserved orthologous genes identified from the MQTL (called ortho‐MQTLs) regions of pigeonpea with four legume crops (pea, French bean, cowpea, and chickpea).

Supporting Information 1
Figure S1 Characteristic features of the identified meta‐QTLs (MQTLs): (a) the number of MQTLs on each linkage group; (b) the number of MQTLs identified for each trait group; (c) average confidence intervals (CIs) for initial QTLs and MQTLs, as well as a fold reduction in CI values after MQTL analysis; (d) number of initial QTLs present in MQTLs; (e) MQTLs shared by different trait groups.

Figure S2 Distribution of candidate genes encoding proteins associated with agronomic traits (154), fertility restoration (36), disease resistance (53), and seed quality traits (7) in different MQTL regions.

## Data Availability

Relevant data are included in this paper and its associated Supporting Information section.
